# Extra-short implants (≤ 6.5 mm in length) in atrophic and non-atrophic sites to support screw-retained full-arch restoration: a retrospective clinical study

**DOI:** 10.1186/s40729-023-00499-7

**Published:** 2023-09-13

**Authors:** Eduardo Anitua, Asier Eguia, Mohammad Hamdan Alkhraisat

**Affiliations:** 1 Private Practice in Oral Implantology, Clínica Eduardo Anitua Foundation, C/ Jose Maria Cagigal 19, 01007 Vitoria, Spain; 2 University Institute for Regenerative Medicine and Oral Implantology, UIRMI (UPV/EHU-Fundación Eduardo Anitua), Vitoria, Spain; 3 BTI Biotechnology Institute, Vitoria, Spain; 4 University of the Basque Country UPV/EHU, Leioa (Bizkaia), Spain

**Keywords:** Short dental implants, Full-arch, Non-atrophic sites, Implant survival, Marginal bone level

## Abstract

**Purpose:**

Increasing scientific evidence support extending the application of short dental implants to non-atrophic dental arches. The purpose of this study has been the evaluation of extra-short implants (≤ 6.5 mm in length) that were placed in atrophic and non-atrophic anatomical sites to support the same prosthesis.

**Methods:**

For that, a retrospective study was conducted by including complete dentures that were solely supported by extra-short implants in the maxilla and/or the mandible. Clinical data about patients, implants, anatomy, and prosthesis were obtained. Statistical analysis was performed to assess implant- and prosthesis-survival, changes in the marginal bone level and prosthetic complications.

**Results:**

A total of 87 implants in 15 screw-retained complete dentures were assessed. None of the prostheses nor the extra-short implant failed during the follow-up of 27.2 ± 15.4 months. The changes in the mesial and distal marginal bone level were + 0.15 ± 0.51 mm and + 0.11 ± 0.50 mm, respectively. Comparing the implants according to the availability of sufficient bone to place longer implants, indicated the absence of significant differences in the changes of the mesial marginal bone level. However, the changes in the distal marginal bone level showed a statistically significant difference in favor of implants that were placed in non-atrophic sites. Two events of screw loosening were reported that were resolved by retightening the screws.

**Conclusions:**

Implant- and prosthesis-related outcomes support the use of extra-short implants in atrophic and non-atrophic site to support complete prosthesis.

**Graphical Abstract:**

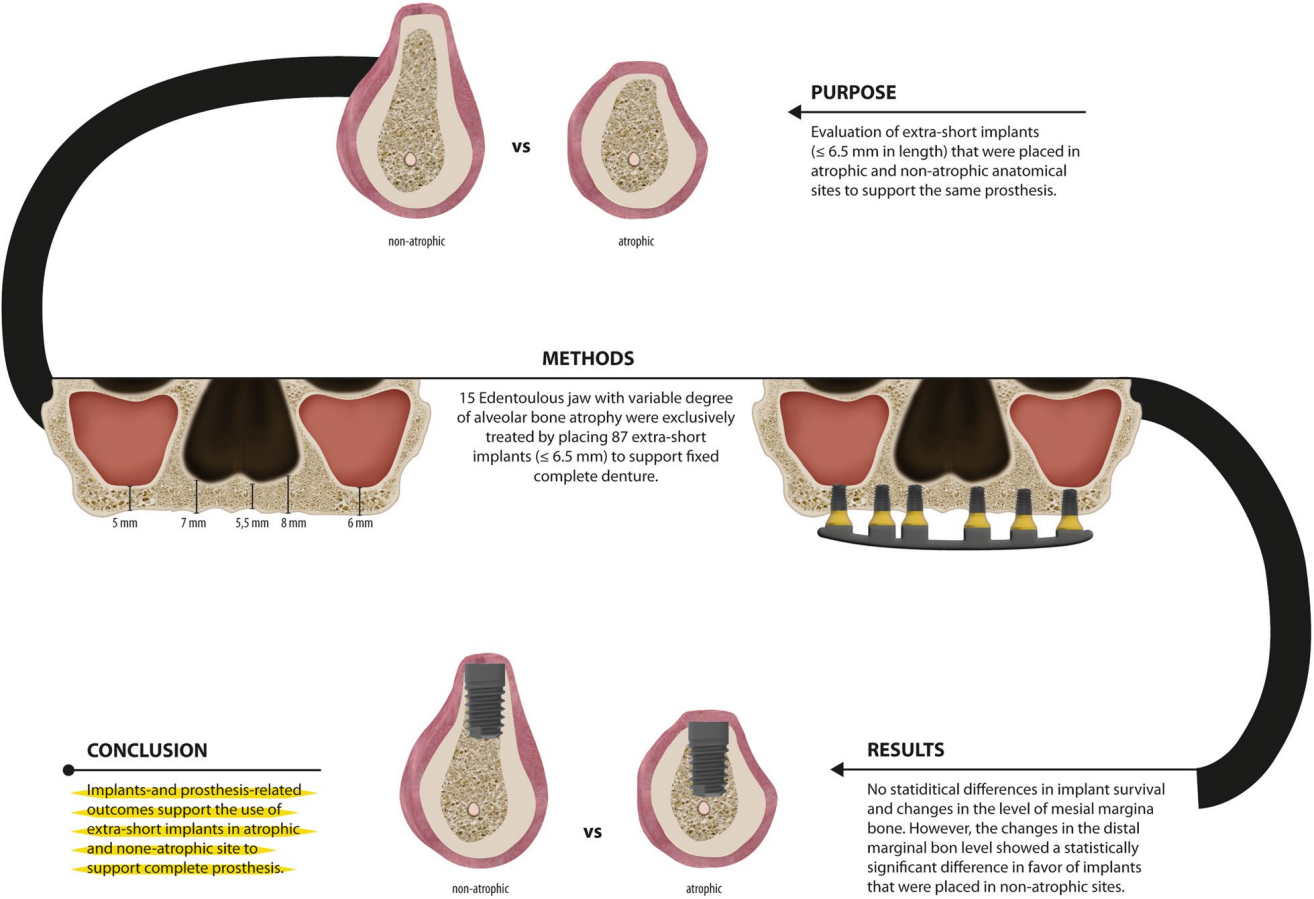

## Background

Edentulism is a risk factor for the stability of the dental arches as it resulted in 3D atrophy of the alveolar process (horizontally and vertically) [1–4]. The consequences of alveolar bone atrophy include shorter face, rotation of the mandible in upward direction and more backward position of the tongue at rest [5]. These changes will not only affect oral functions (mastication and speech) and esthetics but will rather predispose to health-related problems due to obstructive sleep apnea, cognitive impairment, limited food choices and social interaction [5–9].

Alveolar bone atrophy after tooth loss is progressive, cumulative, and irreversible and patients with long-term edentulism would suffer from advanced stages of bone atrophy affecting the stability of mucosa-borne dental prosthesis [1–4]. Implant-supported prosthesis would be a valuable option to improve retention, stability, and function in these patients; however, the presence of alveolar bone atrophy would preclude the placement of dental implants unless a bone augmentation procedure is performed [1, 10]. Patient’s age and medical conditions may advise against performing advanced bone augmentations and would incline the balance toward short and minimally invasive implant surgery. In this context, short dental implants would be the clinician’s best ally.

From clinical point-of-view, the use of short implants has been associated with less biological and surgical complications, surgery time and treatment cost [11, 12]. They simplify the surgical intervention and provide a less invasive alternative to bone augmentation procedures (to place longer implants) [13–15]. Moreover, they show similar outcomes to longer implants in terms of implant survival and marginal bone stability [16–22]. From technological point of view, the advancement in the macro- and micro-design of the dental implants and the prosthetic components have shifted the definition of the short dental implant toward lesser values and the clinical evidence is supporting this change [16–18, 23, 24]. Careful handling of tissues during surgery through the refinement of the surgical techniques has been also an important factor [25, 26]. Nowadays, implant dentistry is 3D-centric in all the phases of treatment (diagnosis, planning and execution). The net outcome of all these advancements is a wider indication of short dental implants to more reduced residual alveolar bone heights [13, 15, 27–29].

The increasing supporting evidence about short dental implants is a good reason not to limit their use to those cases where there is no sufficient bone to place longer implants [18]. There is a clinical claim to compare short and longer implants under similar conditions [30]. Thus, testing short implants in an alveolar ridge where longer implants could be placed. Indeed, several clinical trials have compared the short and long implants in this context [31–40]. Similar outcomes have been reported for short and long implants in terms of implant survival and marginal bone loss. However, additional studies are needed to assess the predictability of using short implants in different scenarios of alveolar bone height [18]. Thus, completely edentulous mandible/maxilla could be an interesting model as the residual alveolar bone height would vary from one anatomical site to another.

Extra-short dental implants (≤ 6.5 mm) [41, 42] have a lower osteointegration surface than long implants and several clinical reports have assessed them [43, 44]. On the short- and medium-term follow-up, no statistically significant differences have been observed between extra-short and longer implants in implant survival or marginal bone-level changes [43, 44].

Thus, the purpose of this retrospective study is to assess extra-short implants (≤ 6.5 mm in length) [41, 42] that were placed in atrophic and non-atrophic anatomical sites to support full-arch restoration.

## Methods

This article was written following Strengthening the Reporting of Observational studied in Epidemiology (STROBE) guidelines [45]. The research has been conducted according to the Declaration of Helsinki and its amendment. It was approved by the ethical committee of Araba University Hospital (FIBEA-02-ER/22/Extracortos).

Pseudonymized electronic database was consulted to retrieve the records of patients with the following characteristics: extra-short implants supporting a fixed complete denture, the use of transepithelial abutment, patients 18 years old or more and a radiographic follow-up of a minimum of 12 months.

### Surgical intervention

The extra-short implants (length ≤ 6.5 mm) were placed following the manufacturer's instructions (UnicCa^®^ implants, BTI Biotechnology Institute, Vitoria, Spain). The bone at the implant site was drilled following the low-speed drilling procedure. The initial drill was operating at 800–1000 rpm with irrigation and the diameter drills were working at low speed (≤ 150 rpm) without irrigation [46]. Before implant insertion, liquid plasma rich in growth factors (PRGF) was placed in the neo-alveolus and the implants were inserted with the aid of a surgical motor at 25 Ncm. The implants were finally seated with a calibrated torque wrench at the level of alveolar bone crest. The PRGF was prepared using an available commercial kit (KMU 15, BTI Biotechnology Institute, Vitoria, Spain) [47, 48].

### Implant loading and prosthetic rehabilitation

The implant loading protocol was decided taking into considerations the insertion torque and the bone type. As such, immediate implant loading was performed for those implants that were inserted at an insertion torque ≥ 25 Ncm and in a bone with good quality.

For loading, definitive transepithelial abutment (Multi-Im^®^) was connected to the implant following the one abutment one time principle. The impression making and the prosthetic rehabilitation were thus performed at the gingival level to deliver a screw-retained complete prosthesis. For the provisional prosthesis, an articulated titanium bar system (BTI Biotechnology, Vitoria, Spain) veneered with resin material was used. The definitive prosthesis was made from a metallic framework that was computer-designed and computer-manufactured. The structure was then ceramic-veneered to deliver the definitive screw-retained prosthesis at mutually protected occlusion.

### Data extraction

The study database was generated by incorporating the following variables:

Principal variable: Implant survival rate.

Secondary variables: Patients’ age and sex, medical history, implant length and diameter, anatomical site, bone type and density at the implant site, residual bone higher (at implant site), insertion torque, bone augmentation surgery, date of implant insertion, date of implant loading, length of the transepithelial abutment, crown-to-implant ratio, type of the antagonist, marginal bone level at loading (mesial and distal), date of the last available radiograph, marginal bone level at the last available radiograph (mesial and distal), technical complications and date of the last visit.

On pre-surgical cone-beam CT scan, the residual alveolar bone height (the vertical distant from the crest to the maxillary sinus, the nasal cavity or the mandibular canal) and the bone density were measured with the help of a software (BTI Scan IV^®^).

**Fig. 1:**
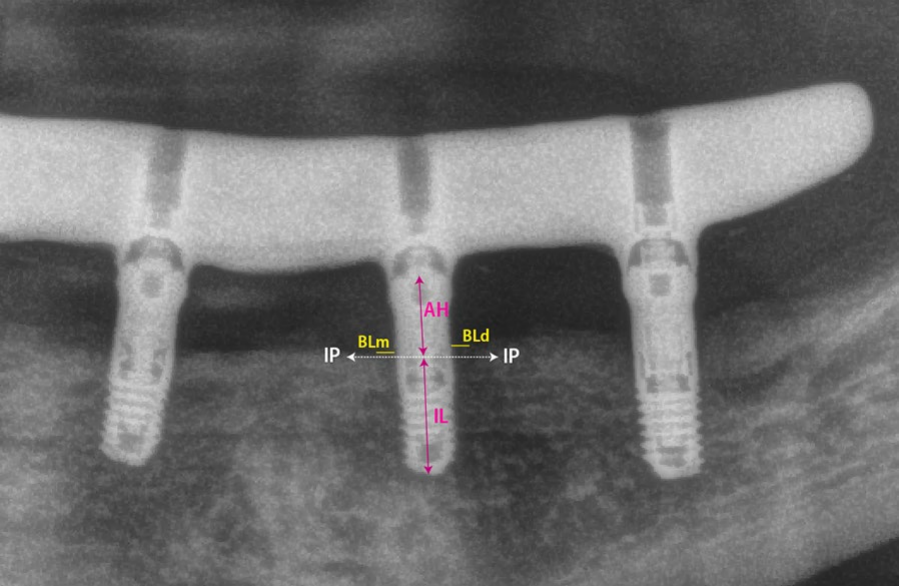
Bone-level measurements. Distances between the IP (implant platform) and the first bone implant contact coronally (BLm, bone level mesial; BLd, bone level distal) were measured and calibrated based on the known lL (implant length). AH (abutment height)

Panoramic radiograph was made by positioning the patients on the chin resting device and orienting the Frankfurt plane to be parallel to the ground. The radiographs at implant loading and the last available one were assessed to determine the marginal bone level. For that, the radiograph was visualized on a dental software (Sidexis; Dentsply Sirona; York, US) where the measurements were calibrated by the known implant length (Fig. 1). The vertical distance between the implant platform and the first coronal bone-to-implant contact was measured both mesially and distally. The measurements had a negative sign if the bone level was below the implant platform and positive sign if it was above the implant platform. The differences in the bone marginal level between the two radiographs determine the change in the bone marginal level. Furthermore, to calculate the crown-to-implant ratio, the crown length was divided by the sum of the lengths of the implant and the transepithelial abutment.

### Statistical analysis

All the statistical analysis was performed in a software package (IBM SPSS Statistic, SPSS Inc., Chicago, IL, USA). Statistical significance was set at *p* value < 0.05. The principal factor was the sufficiency of alveolar bone height to place longer implants than 6.5 mm. Descriptive analysis was performed by calculating the frequency for categorical variables and the mean and standard deviation for the continuous variables. The Shapiro–Wilk test indicated the distribution type (normal or not). Accordingly, the statistical testing of the significant of the differences was either tested by the Mann–Whitney test (bone density, insertion torque, follow-up time and crown-to-implant ratio) or Student’s test (changes in the marginal bone level). For categorical variables, this was done by the Chi-square test.

## Results

### Patients’ characteristics

The analysis was performed in 14 patients (11 women and 3 men) who had a mean age of 70 years (range: 58 to 85 years). Three patients had arterial hypertension, one patient had a pacemaker, and another patient was an active smoker.

### Implants’ characteristics

The study included 15 complete dentures in the mandible and the maxilla that were supported by 87 extra-short implants (37 in the maxilla and 50 in the mandible). Table 1 shows the distribution of the implants’ diameter and length. The 74.7% of the extra-short implants were also narrow implants (diameter ≤ 3.5 mm).

**Table 1:** Distribution of the length and the diameter of the dental implants in the maxilla and the mandible

			Length (mm)			Total
			4.5	5.5	6.5	
Maxilla	Diameter	2.50		3	0	3
		3.00		5	3	8
		3.30		3	3	6
		3.50		2	0	2
		3.75		5	0	5
		4.00		3	3	6
		4.25		0	1	1
		4.75		1	1	2
		5.00		2	1	3
		5.50		1	0	1
	Total			25	12	37
Mandible	Diameter	2.50	0	0	1	1
		3.00	0	2	4	6
		3.30	0	2	18	20
		3.50	1	5	13	19
		3.75	1	1	2	4
	Total		2	10	38	50

The upper dentures were supported by 6 (1 prosthesis), 7 (1 prosthesis) and 8 (3 prostheses) implants. All of them were bilaterally extended to the second molar area. The distribution of the dental implants that were supporting these prostheses is shown in Table 2. All the prostheses had implants that were placed at teeth #11, #17 and #27.

**Table 2:** The distribution of the dental implants in the upper arch for each complete prosthesis

Implant position^a^	Prosthesis					Total
	1	2	3	4	5	
11	1	1	1	1	1	5
12	1	0	0	0	0	1
13	0	0	1	1	1	3
14	1	1	1	1	0	4
15	0	0	0	0	1	1
17	1	1	1	1	1	5
21	0	1	0	1	1	3
22	1	0	1	0	0	2
23	0	0	1	1	1	3
24	1	0	1	1	0	3
25	0	1	0	0	1	2
27	1	1	1	1	1	5
Total	7	6	8	8	8	37

^a^Implant position was defined following the FDI tooth numbering system

**Fig. 2:**
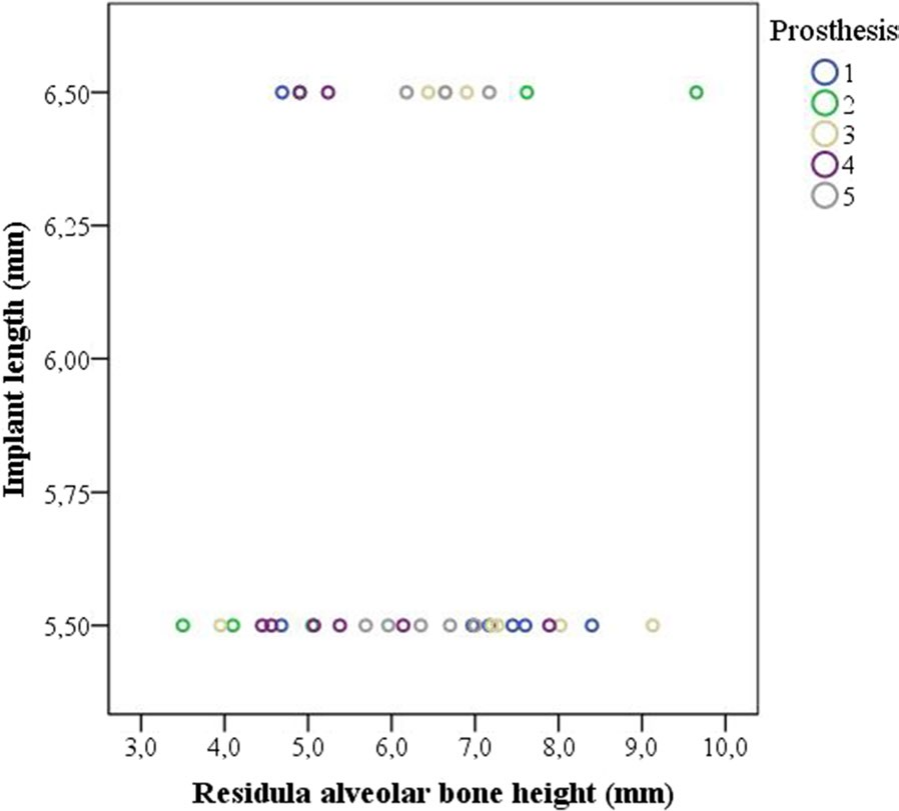
The length of the extra-short implants that were placed at maxillary sites with and without sufficient height to place longer implants

Figure 2 shows that the extra-short implants in the maxilla were placed at sites with and without sufficient height to place longer implants. Longer implants were possible to be placed in 40% of the implant’s sites. Three 5.5 mm long implants were placed simultaneously to transcrestal sinus lift (residual bone height of 3.5, 4.7 and 5.1 mm) and another implant simultaneously to nasal floor elevation (residual bone height of 4.1 mm). Two 6.5-mm-long implants were placed simultaneously to transcrestal sinus lift (residual bone height of 4.7 mm) and nasal floor elevation (residual bone height of 4.9 mm).

**Fig. 3:**
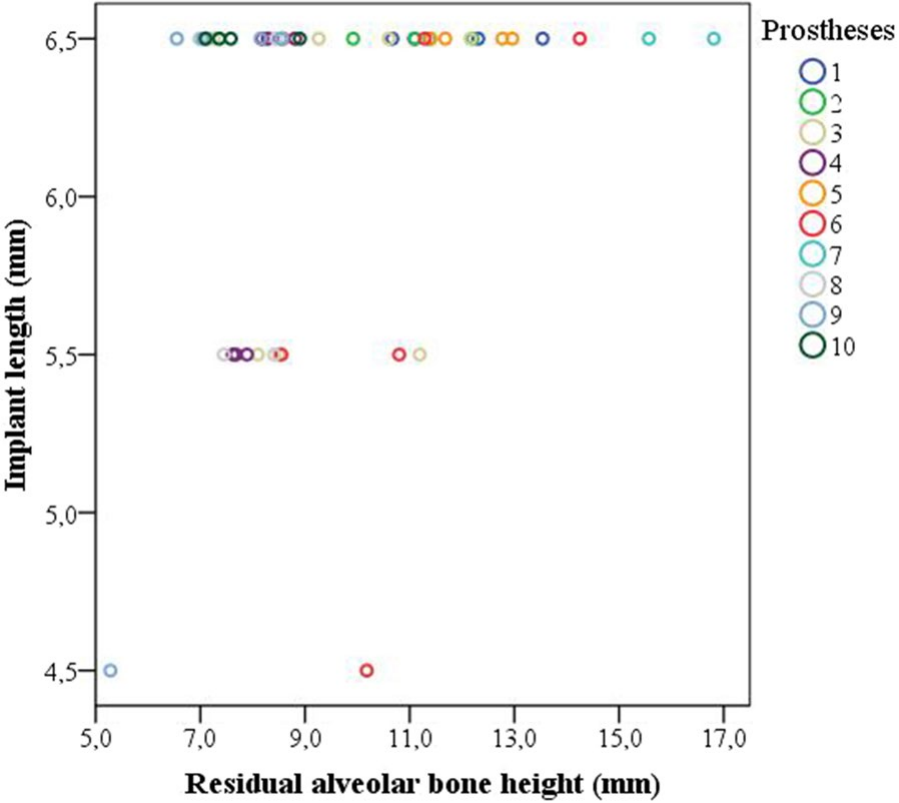
The length of the extra-short implants that were placed at mandibular sites with and without sufficient height to place longer implants

The lower dentures were supported by 4 (5 prostheses), 5 (1 prosthesis), 6 (3 prostheses) and 7 (1 prosthesis) implants. Seven prostheses were extended to the premolar area and another 3 to the molar area. The distribution of the implants in the lower arch is shown in Table 3. The extra-short implants were distributed evenly within the arch in 6 prostheses. Figure 3 shows that 94% of the residual alveolar heights were sufficient to place longer implants than 6.5-mm-long implants.

**Table 3:** The distribution of the dental implants in the lower arch for each complete prosthesis

Implant position^a^	Prosthesis										
	1	2	3	4	5	6	7	8	9	10	Total
32	1	1	1	1	1	1	1	0	1	0	8
33	0	0	0	0	0	0	0	1	0	1	2
34	0	1	1	1	1	0	1	0	1	0	6
35	1	0	1	0	0	1	0	1	1	1	6
36	0	0	1	1	0	0	0	1	0	0	3
37	0	0	0	0	0	1	0	0	0	0	1
41	0	0	1	0	0	0	0	0	0	0	1
42	1	1	0	0	1	1	1	0	1	0	6
43	0	0	0	1	0	0	0	1	0	1	3
44	0	1	1	0	1	1	1	0	0	0	5
45	1	0	0	1	0	0	0	1	1	1	5
46	0	0	1	0	0	0	0	1	0	0	2
47	0	0	0	1	0	1	0	0	0	0	2
Total	4	4	7	6	4	6	4	6	5	4	50

^a^Implant position was defined following the FDI tooth numbering system

### Atrophied and non-atrophic site: surgical and performance outcomes

Data stratification by the presence or absence of sufficient bone height to place longer implants is shown in Table 4. The bone density was significantly higher at the sites with sufficient height to place longer implants. This had an impact on the insertion torque that scored higher values at these sites. Immediate implant loading was performed more frequently in the sites with sufficient bone height (47 out of 63 implants) in comparison with sites of insufficient height (4 out of 24 implants) to place longer implants. These differences were statistically significant (Chi-square test, *p*-value: 0.000).

**Table 4:** Effect of the availability of sufficient bone height to place longer implants on bone density, insertion torque, follow-up time and changes in the marginal bone level and the crown-to-implant ratio

Sufficient height to place longer implant?		Bone density	Insertion torque (Ncm)	Follow-up time (months)	Change in marginal bone level (mm)		CIR^c^
					Mesial	Distal	
Yes (63 implants)	Mean	855	46	27.4	0.17	0.19	2.75
	Median	950	50	24.1	0.10	0.14	2.59
	Range	60 to 1300	5 to 70	11.1 to 67.0	− 0.90 to 1.50	− 1.50 to 1.10	1.30 to 4.70
	Standard deviation	306	15	15.2	0.54	0.48	0.74
No (24 implants)	Mean	531	26	26.7	0.08	− 0.10	2.90
	Median	500	25	16.8	− 0.03	0	2.97
	Range	200 to 950	5 to 65	14.9 to 67.0	− 0.80 to 0.80	− 1.60 to 0.70	1.90 to 3.90
	Standard deviation	159	16	16.2	0.42	0.50	0.57
*p*-value		0.000^a^	0.000^a^	0.475^a^	0.414^b^	0.018^b^	0.153^a^
Total	Mean	766	40	27.2	0.15	0.11	2.79
	Median	800	45	21.0	0.09	0.10	2.80
	Range	60 to 1300	5 to 70	11.1 to 67.0	− 0.90 to 1.50	− 1.60 to 1.10	1.30 to 4.70
	Standard deviation	309	18	15.4	0.51	0.50	0.69

^a^Mann–Whitney test, ^b^Student’s test, ^c^crown-to-implant ratio

**Fig. 4:**
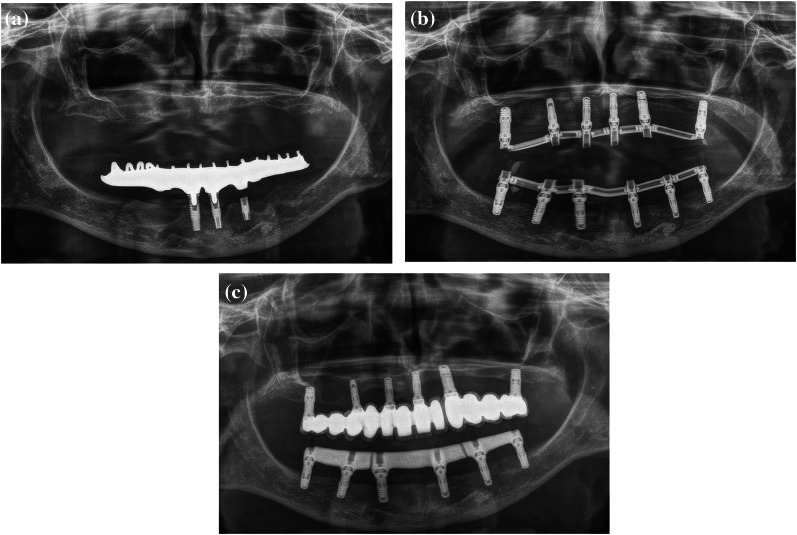
Clinical case. Full-arch mandibular rehabilitation on 6 narrow (≤ 3.5 mm) extra-short (≤ 6.5 mm) implants.** A** Initial situation. Previous fixed full-arch lower maxillary implant rehabilitation on 4 standard-length implants failure. **B** Provisional prosthesis screw-retained on intermediate abutments (transmucosal abutments), reinforced with metal bars and resin veneered. **C** Definitive prosthesis screw-retained on intermediate abutments. CAD-CAM metal suprastructure split in three sections. Implant diameter and length (mm): #4.7 (3.5 × 5.5), #4.5 (3.5 × 5.5), #4.3 (3 × 6.5), #3.2 (3 × 6.5), #3.4 (3 × 6.5), #3.6 (3.3 × 5.5)

**Fig. 5:**
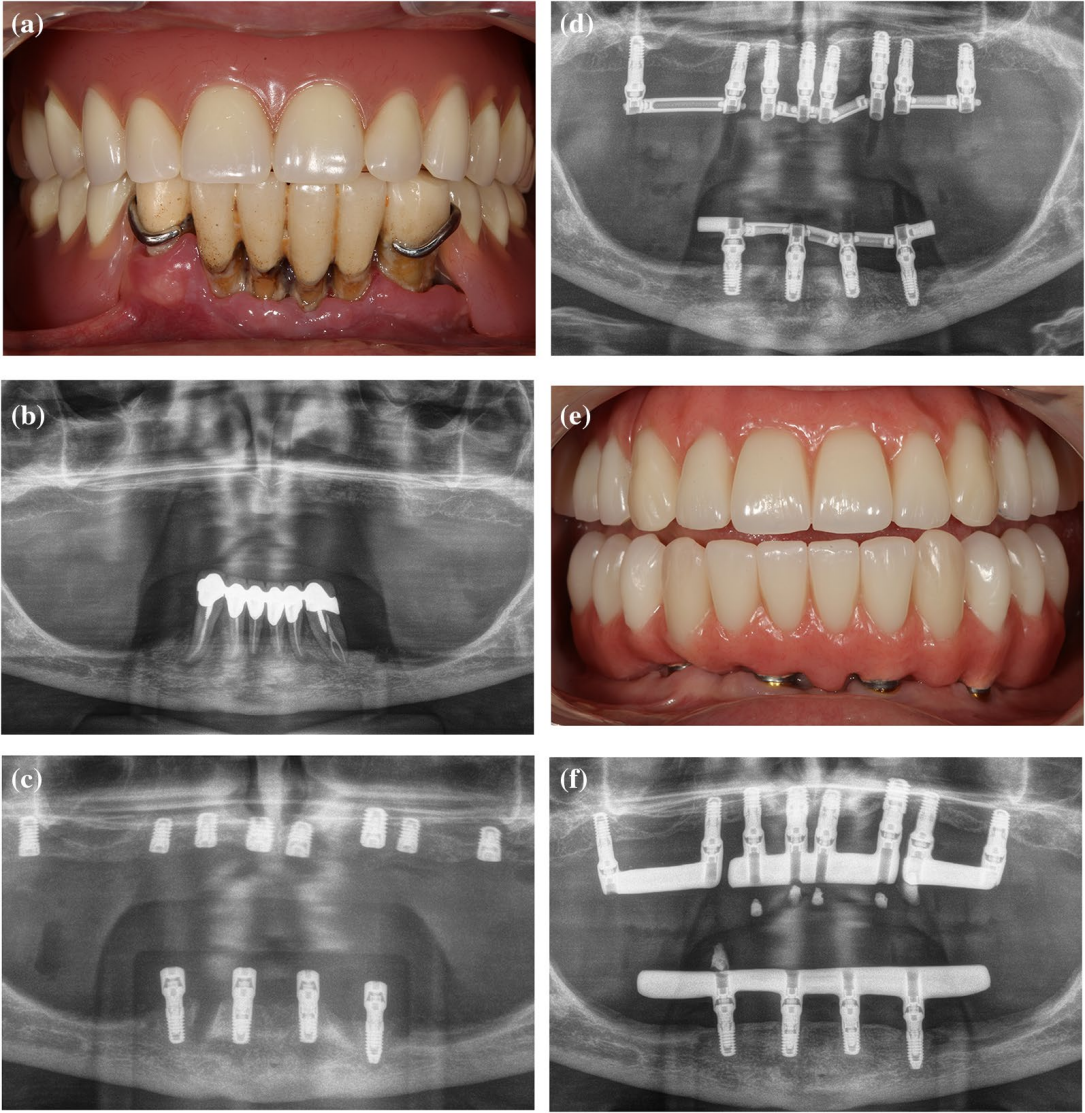
Clinical case. Full-arch maxilla rehabilitation on 8 extra-short (≤ 6.5 mm) implants.** A** Clinical picture showing removal complete denture in the maxilla. **B** Extra-oral radiograph showing the initial situation of the completely edentulous maxilla. **C** Placement of 8 dental implants in the maxilla. Implant diameter and length (mm): #1.1 (3.75 × 5.5), #1.3 (3.3 × 5.5), #1.4 (3 × 5.5), #1.7 (3 × 6.5), #2.1 (3.5 × 5.5), #2.3 (4 × 6.5), #2.4 (3 × 5.5) and #2.7 (3.5 × 5.5). **D** Provisional prosthesis screw-retained on intermediate abutments, reinforced with metal bars and resin veneered. **E** Clinical picture of the definitive prosthesis screw-retained on intermediate abutments. **F** Follow-up after 31 months of implant insertion

None of the implants failed during the follow-up of 27.2 ± 15.4 months. Figures 4 and 5 show clinical cases of completely edentulous arch that were treated by the insertion of extra-short implants. The changes in the mesial and distal marginal bone level were + 0.15 ± 0.51 mm and + 0.11 ± 0.50 mm, respectively. There were no significant differences in the follow-up time and the mesial marginal bone-level changes regarding the availability of sufficient bone to place longer implants (Table 4). However, the changes in the distal marginal bone level showed a statistically significant difference.

Table 5 shows length of the transepithelial abutments in relation to the implant length. The most frequent lengths of this prosthetic component were between 2.5 and 4.0 mm. None of the prostheses failed and only 2 showed technical complications. These were screw loosening in two lower dentures that occurred once and were resolved by screw retightening.

**Table 5:** The length of transepithelial abutments in relation to the length of the implant

Transepithelial abutment length (mm)	Implant length (mm)			Total
	4.5	5.5	6.5	
1.0	0	1	1	2
1.5	0	2	0	2
2.0	1	1	0	2
2.5	0	3	11	14
3.0	0	7	16	23
3.5	0	9	8	17
4.0	0	10	13	23
5.0	1	2	1	4
Total	2	35	50	87

## Discussion

There is an interest and a need to assess the use of short implants not only in atrophic alveolar process, but also in those where sufficient bone height is available to place longer implants. This retrospective study is reporting on the use of extra-short implants in these two situations. Fifteen screw-retained fixed complete prostheses have been supported by 87 extra-short implants (length ≤ 6.5 mm). Sixty-three implants have been placed at sites where longer implants could be placed. Promising outcomes (implant survival, marginal bone stability and technical complications) could be observed.

Regarding implant survival, several randomized clinical trials (RCTs) have been conducted to compare short implants and long implants when sufficient bone height has been available to place long implants [31–40]. The short implant has been 4 to 6 mm in length and the comparator implant has lengths between 8.5 mm and 11 mm. There have been no significant differences between the two implant types in the survival rate [31–40, 49]. The follow-up time is an important factor to consider as the 5-year survival rate of short implants has been lower than longer implants [50]. Fort that, a recent meta-analysis has pooled the data of implant survival in different follow-up times [18]. The pooled risk ratios of implant survival have been 0.98 (95%CI: 0.96 to 1.00), 0.98 (95%CI: 0.95 to 1.02) and 0.98 (95%CI: 0.94 to 1.01) at 1-, 3- and 5-year follow-up [18]. These data agree with the outcomes of this study by showing high implant survival rate.

The high implant survival rate are related to the use of dental implants with roughened surface and threaded design that would reduce the risk of osseointegration failure [31–40]. Rocci et al. have reported in a RCT better outcomes for rough-surface implants in comparison to machined-surface implants after 9 years of follow-up [51]. Moreover, short implants with machined surface haven been associated with higher risk of failure even at short follow-up time (3 years) [52, 53]. Primary implant stability is a mechanical outcome that represents the quality of implant fixation in the alveolar bone [46]. It is the interaction between implant design, bone quality and drilling protocol. Achieving a good primary stability is a common clinical parameter to decide on immediate/early loading protocol. For example, insertion torques higher than 35 Ncm or between 20 and 45 Ncm have been recommended to perform immediate loading [54, 55]. Moreover, all these variables (rough surface, good primary stability and threaded implant) would reduce the risk of micromovement and thus osseointegration failure [46, 56–58]. Indeed, several RCTs have compared short and long implants placed in non-atrophic alveolar bone under immediate/early loading protocols at follow-up times of 1 [31, 35, 49], 3 [40], 5 [32, 36–38], and 10 years [39]. Both implant types have generally shown high survival rate with no statistically significant differences. Most of the comparisons that have been performed in alveolar ridge with sufficient bone height to host long implants have been performed in the context of multi-unit prosthesis [31, 32, 34–37, 39, 40]. Splinting dental implants, as in this study, have several advantages from biomechanical point of view: reduction of lateral forces, enhanced distribution of the stress and lowering the stress received by the implant [59–61]. Moreover, the delivery of the prosthesis with a mutually protected occlusion would decrease the stress on the implants [62]. The number and the distribution of the dental implants have been planned to avoid distal cantilever extension. This type of design would provide better prosthesis support, lower stress (implant, abutment and bone) and stress distribution over greater area [63, 64]. All these measures have together resulted in reducing the risk of late implant failure although 74.7% of the extra-short implants have been also narrow implants (diameter ≤ 3.5 mm). Two other clinical studies have shown a high survival rate (93.4% and 100%) and good marginal bone stability in short-term follow-up [65, 66]. However, more studies are warranted to critically assess the clinical performance of short and narrow dental implants.

In this study the changes in the marginal bone level have been + 0.15 and + 0.11 on the mesial and distal sides, respectively. Limited marginal bone loss (mean < 0.5 mm) has been reported for short implants that have been placed in non-atrophic alveolar bone [36, 38, 49]. The meta-analysis by Guida et al. has reported a pooled mean difference in marginal bone-level changes for short and long implants placed in non-atrophic bone of 0.11 (95%CI: − 0.10 to 0.31), − 0.09 (95%CI: − 0.24 to 0.05) and 0.19 (− 0.06 to 0.45) at 1-, 3- and 5-year follow-up [18].

All the implants in this study have been restored by first connecting a definitive transepithelial abutment to the implant and second connecting the prosthesis to the abutment. For that, the stability of the marginal bone level could be related to the effect of one-abutment one time and tissue-level restoration [67, 68]. Moreover, the length of the transepithelial abutments has been ≥ 2 mm for most of the implants (95.4%). It has been reported that abutment height of 2 mm has been associated with minimal marginal bone loss [69]. These abutments have been prefabricated and would have positively affect the sealing quality at the implant–abutment interface against microorganism accumulation and inflammation [70]. Moreover, it will affect the stress transmitted to the bone and avoid excessive stress that would compromise bone stability [71, 72]. The deliver of screw-retained restoration has avoided the risk of residual cement in the soft tissue.

The crown-to-implant ratio is another variable that is increased when extra-short implants are placed (a mean of 2.79 in this study), however it has not influenced the clinical outcomes [73]. This is in agreement with a meta-analysis of single-tooth implants (more vulnerable than splinted implants) which concluded that increased crown-to-implant ratio has not incremented the biological or the technical complications [74]. All the complete prostheses, in this study, have survived and only 2 screw loosening events have been observed. These events could be related to inappropriate screw tightening by applying lower torque than the torque recommended by the manufacturer. As once retightened, no more events have been observed [75, 76].

This study is limited by its design (retrospective) and the absence of a control group (long implants). However, it has provided homogenous scenario to assess the use of extra-short implants in atrophic and non-atrophic alveolar bone sites. Moreover, all the implants have been loaded by the same type of the prosthesis and the same method of fabrication. A retrospective study would assess the medical devise in a real-world environment, reflecting the clinical practice. In this study, 87 extra-short implants have been assessed. The trial sequential analysis has indicated the need for more clinical studies that compared short and long implants in non-atrophic alveolar bone [18]. The meta-analysis has a total size of 916 implants and the required information size has been 1804 implants. The included clinical trials have a variable sample size of the short implants group, it has a wide range between 21 and 121 implants [18]. Longer follow-up time is required to assess the medium and long-term outcomes of short dental implants in non-atrophic alveolar sites.

## Conclusions

The placement of extra-short implants (≤ 6.5 mm) in atrophic and non-atrophic anatomical sites has resulted in similar clinical outcomes (high implant survival and marginal bone stability). The low incidence of complications and the high survival rate of the prosthesis (screw-retained fixed prosthesis) support this extended use of extra-short implants. More clinical studies are needed to offer a reliable clinical indication of short implants in non-atrophic alveolar bone.

## Data Availability

The datasets used and/or analyzed during the current study are available from the corresponding author on reasonable request.
